# Multi-Task Segmentation and Classification Network for Artery/Vein Classification in Retina Fundus

**DOI:** 10.3390/e25081148

**Published:** 2023-07-31

**Authors:** Junyan Yi, Chouyu Chen

**Affiliations:** Department of Computer Science and Technology, Beijing University of Civil Engineering and Architecture, Beijing 100044, China; yijunyan@bucea.edu.cn

**Keywords:** A/V classification, attention, convolution, feature fusion

## Abstract

Automatic classification of arteries and veins (A/V) in fundus images has gained considerable attention from researchers due to its potential to detect vascular abnormalities and facilitate the diagnosis of some systemic diseases. However, the variability in vessel structures and the marginal distinction between arteries and veins poses challenges to accurate A/V classification. This paper proposes a novel Multi-task Segmentation and Classification Network (MSC-Net) that utilizes the vessel features extracted by a specific module to improve A/V classification and alleviate the aforementioned limitations. The proposed method introduces three modules to enhance the performance of A/V classification: a Multi-scale Vessel Extraction (MVE) module, which distinguishes between vessel pixels and background using semantics of vessels, a Multi-structure A/V Extraction (MAE) module that classifies arteries and veins by combining the original image with the vessel features produced by the MVE module, and a Multi-source Feature Integration (MFI) module that merges the outputs from the former two modules to obtain the final A/V classification results. Extensive empirical experiments verify the high performance of the proposed MSC-Net for retinal A/V classification over state-of-the-art methods on several public datasets.

## 1. Introduction

In the field of fundus images analysis, since the retina vessel structures are the only internal human vascular structures that can be observed noninvasively, the research on the segmentation and classification of retina vessels plays an essential role in diagnosing some ocular diseases, such as glaucoma [1], which could lead to blindness. Moreover, many clinical studies have demonstrated that some systemic diseases can lead to irreversible changes in the morphology of the fundus vasculature. For instance, diabetes may cause the growth of many new blood vessels which do not develop properly and can leak easily [2]; systemic arteriolar stenosis and arteriovenous nicking may be caused by long-term hypertension [3]; and the growth of unwanted, abnormal blood vessels in the ordinarily avascular macular area is attributed to Age-related Macular Degeneration (AMD) [4]. Generally, experienced physicians can predict and diagnose some diseases with these changes, and sometimes the different states of the A/V may contribute to this process. Therefore, the quality results of vessel segmentation and A/V classification can help to speed up the diagnosis of some diseases. Nevertheless, manual operations in fundus image regions are time-consuming and labor-intensive, making automatic segmentation and classification methods highly desirable in clinical practice.

Automatic retina A/V classification has been extensively studied with the development of deep learning algorithms. Generally, these methods could be roughly classified into the following two categories, convolution-based and attention-involved algorithms. In the past decade, convolution-based methods have succeeded tremendously by commonly using a series of feature extractors to extract hierarchical information. However, due to the receptive field limitation of pure convolution, these methods yield weak performance for the target structures that show huge inter-patient variation, such as the different shapes and sizes of vessels. For the attention-involved methods, due to the powerful capability of the weight matrix in global context modeling, these methods demonstrate superior performance in improving the overall cohesiveness of the segmented target. Nevertheless, with the poor extraction of short-range dependencies, these methods encounter difficulties in feature extraction at the intersection areas of vessels.

Recently, although many methods have been proposed to reduce the inadequacy caused by the disadvantages, A/V classification is still a challenging task. First, due to the complex environment of the human eyeball, the vessels have highly variable structures, and their morphology is also variable. At the same time, this challenge is accompanied by the high degree of bending of the blood vessels [5], which makes the related challenges more difficult to deal with. Second, tiny peripheral vascular differences between arteries and veins make the classification of arteries and veins more challenging. Especially at the end of blood vessels, the difference between them is even smaller.

To alleviate the above problems, we propose a novel deep learning end-to-end method called the Multi-task Segmentation and Classification Network (MSC-Net) for A/V classification to mitigate the abovementioned limitations, which splits the whole A/V classification task into three subsections, including segmenting the vessels, classifying the arteries and veins, and feature fusion. First, a Multi-scale Vessel Extraction (MVE) module is proposed to solve the first subtask, vessel segmentation. To enlarge the receptive field of the convolution-based method, we utilize a group of tiny-kernel convolution blocks for replacing the single convolution layers in the U-like architecture, making the outputs of the proposed module obtain the information from all stages. This approach enables the module to extract the detailed features of vessels efficiently. After that, a novel attention-based, Multi-structure A/V Extraction (MAE) module is proposed to address the second subtask. Due to the powerful capability of extracting the long-range dependencies between pixels, this module could easily obtain information about differences between pixels, which is highly advantageous for accurately distinguishing the arteries and veins. In addition, we combined transformer-like and U-like architectures to obtain the features from different scales. To reduce the computational cost, we adjusted the strategy of obtaining global contextual attention, avoiding the issue of binary complexity that could arise from the matrix operations. Finally, a newly designed Multi-source Feature Integration (MFI) module is proposed to complete the final subtask. Compared with the traditional feature fusion module, the proposed module could capture the detailed information of vessels to optimize the A/V feature. The features of vessels and A/V come from different sources and have different characteristic distributions. With the help of this module, the network could suppress background-prone features to pay more attention to vessel features. In summary, the main contributions are listed as follows:

1. We propose a multi-task segmentation and classification network (MSC-Net) for artery and vein classification in the retina fundus. The highly optimized Python implementations of our method will be released at: https://github.com/chenchouyu/MSC-Net (accessed on 7 May 2023).
2. Novel strategy of A/V classification is proposed, which splits the whole task into three parts, including extraction of vessel features, classifying of artery and vein, and tuning. And we propose three modules to deal with the corresponding tasks.
3. Extensive and comprehensive experimental results on several public datasets demonstrate the validity of novel strategy and MSC-Net for accurate A/V separation and superior performance over existing state-of-the-art methods.

The remainder of this paper is structured as follows: related works are reviewed in Section 2; the detailed description of our network is presented in Section 3; the experimental results, which include the ablation study and comparison experiments, are summarized in Section 4; and finally, the conclusion is presented in Section 5.

## 2. Related Work

Presently, a massive number of deep learning networks for retinal A/V classification have been proposed. These methods could be divided into two categories, including the convolution-based networks and the attention-involved method. For the first category, depending on whether the structural connection or topology is used or not, the conventional methods can be further categorized into hybrid and pure methods. And the second category, according to the composition of the attention module, can be divided into traditional methods and transformer-based methods.

### 2.1. Convolution-Based Methods

Since the proposal of U-Net [6] in 2015, the pure-convolution methods had shown superior capability in dealing with medical image processing. To alleviate the impact of the limited receptive field of convolution operation, the dilated convolution is applied in Chen et al. [7]. This network uses atrous convolutions with different dilation rates to generate multi-scale feature maps, which are combined to capture contextual information at different scales better. Qiangguo et al. [8] proposed a DU-Net that utilizes the deformable convolutional blocks [9] to construct the U-like architecture, demonstrating that the network can capture vessels of various shapes and scales through the deformable receptive fields. This property makes it more suitable for segmentation tasks with irregular structures. The deformable convolution plays a crucial role in achieving these results. Moreover, the inception architecture [10] confirms that the broader structure could provide a larger receptive field. Through this property, AV-Net [11] proposes a novel network established on the inception architecture, showing the strong capability for extracting detailed features. Galdran et al. [12] formulate the classification of arterial and venous vessels as a four-class semantic segmentation problem. This strategy allowed the network to classify vessels as background, artery, vein, or uncertain by considering the inherent uncertainties in the task. Xu et al. [13] further improved the A/V classification by introducing a modified fully convolutional network architecture for arteries and veins segmentation.

Furthermore, some researchers perform the A/V classification tasks in the retina fundus by introducing the structural information into the pure convolution to alleviate the loss of the information and enlarge the receptive field, called the hybrid method. Mishra et al. [14] proposed a novel method, which transforms the vessel feature from the image domain into a graph representation, utilizing the vessel topology to enhance the artery/vein classification. In [15], the retina vessel skeleton, which represents the direction of vessels, is extracted from different color spaces. This information is utilized to alleviate the disconnection in the result. Estrada et al. [16] present a novel method, which combines the tree topology estimation framework with domain-specific features to construct a global likelihood model that is highly effective in classifying A/V content. Zhao et al. [17] developed a novel approach that adapts the concept of dominant set clustering to address the retinal blood vessel topology estimation and A/V classification.

Despite the impressive performance achieved by convolution-based networks in retinal vessel segmentation and A/V classification, the local and limited receptive field of convolution remains a significant challenge for semantic segmentation. To address this issue, the MVE module was proposed. This module replaces the single convolution layer in the U-like architecture with a sequence of small convolution layers, thereby enabling each kernel to extract information from all the feature maps and expand the receptive field of the network.

### 2.2. Attention-Involved Methods

According to the composition of the attention module, they can be divided into traditional methods and transformer-based methods. The SE-Net [18] proposes a novel module to tackle the issue of exploiting channel dependencies. The SE block could squeeze the feature maps from the spatial domain and expand the channel attention to the global weight matrix. Inspired by the SE block, Xiang et al. [19] proposed a SE-based block called SK block, which employs an inception-like architecture to fuse the feature maps from multi-branch, and obtains the detailed channel attention matrix. Jun et al. proposed a novel dual attention mechanism that utilizes both spatial attention and channel attention to fuse the final attention representation, which achieves scene segmentation by manipulating different levels of features. In another paper [20], the authors proposed an attention mechanism called the Bottleneck Attention Module (BAM), which integrates global and local information via a bottleneck structure and channel attention mechanism. The BAM can automatically learn to selectively emphasize informative features and suppress redundant features by adaptive feature recalibration.

Since the proposal of Transformer [21] in 2017, designed for Natural Language Processing, more and more transformer-like architectures have been presented for the image processing field. For instance, Vision Transformer (ViT) [22] is the first work that utilizes the transformer-like architecture for replacing standard convolutions in deep neural networks. The original transformer processes the ’patches’ of an image to obtain the correct categories. Thanks to the robust global context extraction, the network could easily establish the long-range dependencies of pixels, which is beneficial to downstream tasks, such as classification and segmentation. To reduce the computational cost, the MixFormer [23] devises an asymmetric attention scheme, which handles multiple target templates. For accelerating the network, Yehao Li et al. [24] introduced a novel Contextual Transformer (CoT) block for visual recognition, which fully capitalizes on the contextual information among input keys to guide the learning of the dynamic attention matrix. Liu et al. presented a Shifted windows (Swin) Transformer [25] that utilizes a shifted window along the spatial dimension to model global and boundary features, in which the feature maps, *Q*, *K*, and *V*, are obtained by a smaller size compared to the ViT. Although the transformer has the advantage of global receptive field capture capability, the expensive calculation may limit its application in medical image processing tasks. To tackle this issue, the MAE module is proposed in this work. This module hybrids the transformer-like blocks and a U-like architecture to generate different scale feature maps and control the computing cost, efficiently.

## 3. Multi-Task Segmentation and Classification Network (MSC-Net)

**Figure 1:**
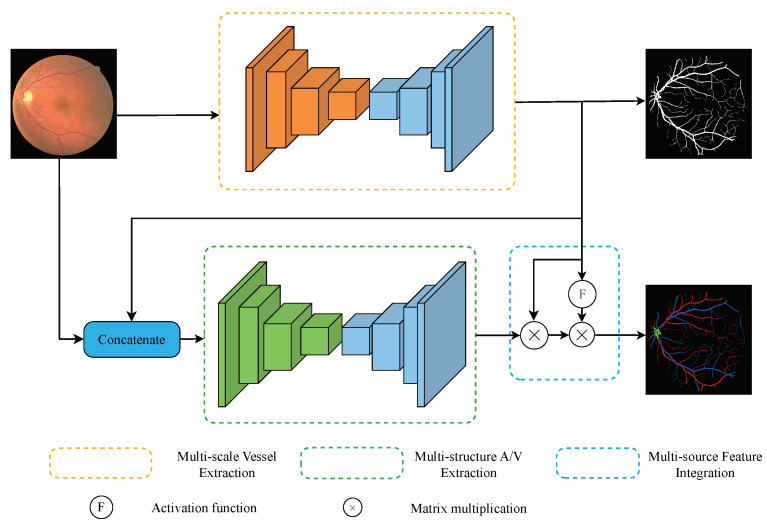
The structure of MSC-Net.

To alleviate the challenges in A/V classification, we propose a novel Multi-task Segmentation and Classification Network (MSC-Net). The process of MSC-Net is illustrated in Figure 1.

The MSC-Net consists of three modules: the Multi-scale Vessel Extraction (MVE) module, the Multi-structure A/V Extraction (MAE) module, and the Multi-source Feature Integration (MFI) module. First, in the MVE module, the input image is operated by a full convolution network. The semantic and detailed structural information of vessels is extracted by groups of the tiny-kernel convolution layers and reorganized to establish a more accurate tree-like vessel map. Then, in the MAE module, the concatenation of the original image and the output of the MVE module is handled by a hybrid architecture. This module obtains better perception capability of long-term modeling by adding the attention operation, making it more robust in dealing with minor differences. Finally, the A/V feature maps extracted by the MAE module and the vessel features extracted by the MVE module are fed to the MFI module, which fuses the detailed information of the two aspects to obtain the final classification results, demonstrating that the second introduction of vessel information is beneficial to the A/V classification. In the following sections, the novel modules in MSC-Net will be described in detail.

### 3.1. The Multi-Scale Vessel Extraction (MVE) Module

**Figure 2:**
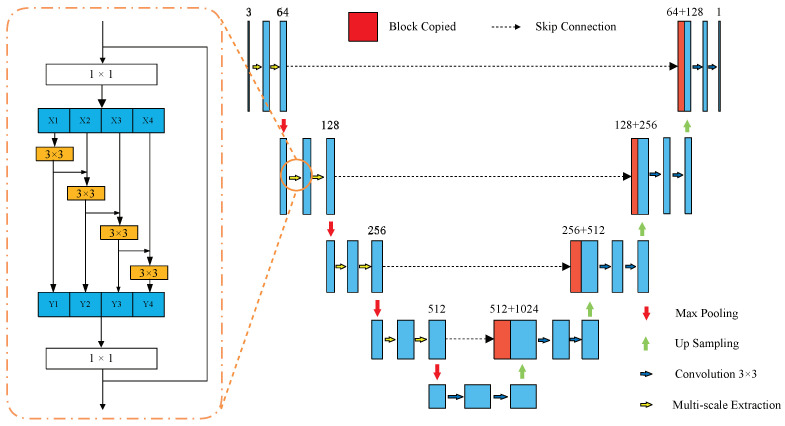
The structure of the MVE module. The subfigure on the left shows the detailed structure of the Multi-scale Extract, which is labeled with the yellow arrow in the left subfigure.

The task of A/V classification is divided into three subtasks, including vessel segmentation, A/V classification, and results fine-tuning. We introduce the MVE module to address the first subtask of A/V classification. The MVE module is established on a U-like architecture, using specially adjusted convolution blocks to obtain the multi-scale feature representations. Detailed information about the MVE module is shown in Figure 2.

As a U-like architecture, the MVE module consists of a symmetric encoder and decoder. And the output of each stage of the encoder would be transferred to participate in the operation in the corresponding stages in the decoder. In the encoder of this module, we utilize the Res2Net-like structure for replacing the single convolution blocks which performed as the encoder stages in the original U-Net. The detailed structure of the Res2Net is shown as the right subfigure in Figure 2. Inspired by the Res2Net [26] architecture, we utilize a group of small convolution blocks, signed as $F_{i}\left(*\right)$, to deal with the feature maps extracted by a convolution layer with a kernel size of 1 × 1. The feature map (*x*) would be divided into several subsets, described as $x_{i}$, where *i* refers to the sequence number of a subset, illustrating the different feature representations in channels. Subsequently, except the first subset, each subset ($x_{j}$) would concatenate with the output ($y_{j-1}$) of the small convolution block ($F_{j-1}\left(*\right)$) which processes the pervious subset ($x_{j-1}$). Then, the concatenation would be transferred to $F_{j}\left(*\right)$ to obtain $y_{j}$, which represents the detailed extracted features of different channels. This process can be described as follows:

(1) $$y_{i}=\left\{\begin{array}{cc} x_{i}, & if\;i=1\\ F_{i}\left(\left(x_{1};y_{i-1}\right)\right), & if\;1<i\leq n \end{array}\right.$$

Through this approach, each $F_{i}\left(*\right)$ could obtain information from all feature stages. The new feature extraction strategy obtains a larger receptive field than single convolution. In this module, the feature maps obtained by the 1 × 1 convolution layer are split into four subsets. With this special connection mode, the amount of each block parameter is declined by approximately four times.

### 3.2. The Multi-Structure A/V Extraction (MAE) Module

**Figure 3:**
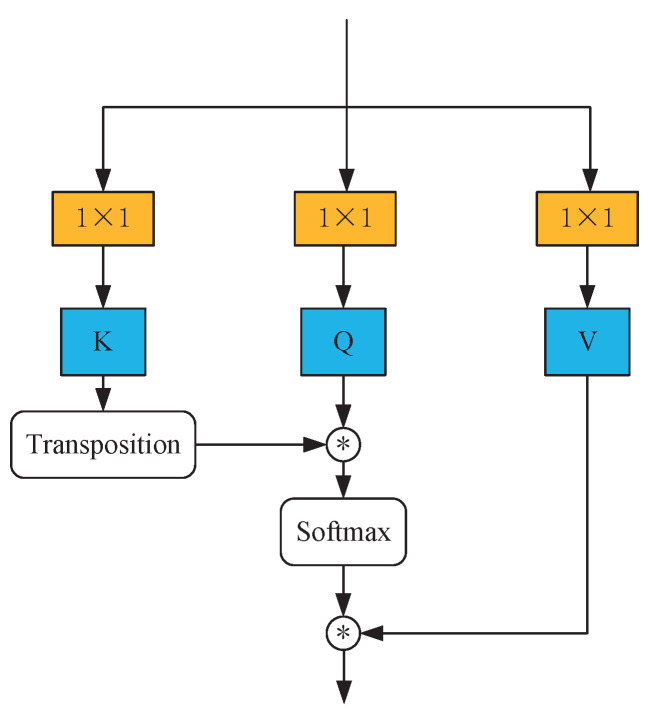
The main block of transformer. In this block, the features are produced by the former modules. ⊛ denotes the local matrix multiplication.

The task of obtaining the characteristic expression of arteries and veins (A/V) is more challenging compared with the extraction of vessel structural features. A more robust feature extraction capability of the network is required for addressing the task. In computer vision, applying the attention mechanism has enabled researchers to value the solid global modeling ability it provides. As a result, more attention-based networks have been proposed. One such network is the Vision Transformer (ViT), introduced in 2021, which can efficiently learn and understand the global dependencies between structures, as shown in Figure 3. In this method, the after-embedded feature maps would be divided into three groups, called keys (*K*), quires (*Q*), and values (*V*), by fully connected layers. Then, the output of the attention machine could be calculated as (2), where $K^{T}$ represents the transposition of keys (*K*), $d_{k}$ refers to the dimension of keys (*K*) and queries (*Q*).

(2) $$Output=softmax(\frac{QK^{T}}{\sqrt{d_{k}}})V$$

**Figure 4:**
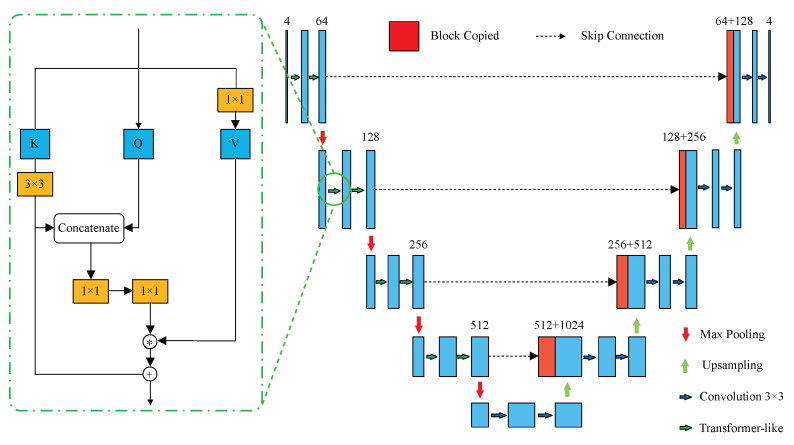
The structure of the MAE module. The subfigure on the left shows the detailed structure of a transformer-like block.

However, due to the high computation cost and the lack of multi-scale feature inputs, ViT is unsuitable for pixel-level classification. To address these limitations and obtain a more reasonable A/V representation, we introduce the Multi-structure A/V Extraction (MAE) module, which consists of transformer-like and convolution blocks that could efficiently extract feature maps of A/V. The detailed architecture of the MAE module is shown in Figure 4. By adding a U-like structure, the transformer-like blocks can obtain multi-scale features, which is beneficial for extracting features of targets with large-scale differences. Additionally, we replace the matrix multiplication between *Q* and *K* with concatenation operations, avoiding the binary complexity problem.

As shown in the right subfigure in Figure 4, the input data in the transformer-like block is first processed by a convolution layer with a kernel size of 3 × 3, resulting in the output *X*. Then, the value (*V*) is computed via $W_{v}$, just like in ViT. However, instead of computing queries (*Q*) and keys (*K*) using extra convolution layers ($W_{q},W_{k}$) as in the typical transformer-like block, we directly transfer *X* into *Q* and *K*. Next, a convolution layer with a large kernel size is used to process *K*, obtaining the static feature representation of the input features, which reflects the information among local neighbors of *K*. Then we employ two convolution layers with a kernel size of 1 × 1 and a soft-max operation to process the concatenation of static representations and *Q* to obtain the weight matrix, which is used to compute the dynamic feature representation via the multiplication between *V* and the weight matrix. We compute the output of this transformer-like block as the sum of the static and dynamic representations, which can be described by Equation (3).

(3) $$Output=K_{s}+V⊛softmax\left(\left[K_{s},Q\right]W_{\theta }W_{\delta }\right)$$

where the ⊛ refers to the matrix multiplication operation, the matrix $W_{\theta }$, and $W_{\delta }$ refer to the two 1 × 1 convolution layers in which the Relu function activates the first one, and the second one is not activated. The $K_{s}$ refers to the static context representation. With the addition of the optimized transformer, the results of each small encoding block obtain enlarged receptive fields compared with the pure convolution block in U-Net.

### 3.3. The Multi-Source Feature Integration (MFI) Module

Due to the fusion of the structural features of the vessel, the performance of A/V classification heavily depends on the accurate extraction of vessel features. Therefore, the incorrect and fractured vessel features impact the coherence of the A/V results, which is a limitation puzzling our method. To alleviate the above challenge, we propose a novel feature fusion module named the Multi-source Feature Integration (MFI) module. In this module, the detailed vessel features obtained by the MVE module are utilized to improve the A/V classification results.

As a binary segmentation task, the extracted vessel feature map before operated by the activation function, the pixels of thick vessels usually obtain a probability close to 1 and the background pixels close to 0, while the boundaries and micro-vessel areas are around 0.5. To enhance the expression of these pixels, we propose a specific activation function, defined as (4). As shown in Figure 1, the vessel maps produced by the MVE module are first processed by the abovementioned activation function. And then, the activated vessel features with more detailed information on the vascular boundaries and microvascular areas are employed to operate the A/V classification results produced by the MAE module.

(4) $$F\left(x\right)=e^{-|x-0.5|}-e^{-0.5}$$

where *x* refers to the probability map of the vessel segmentation, which is produced by the MVE module.

**Figure 5:**
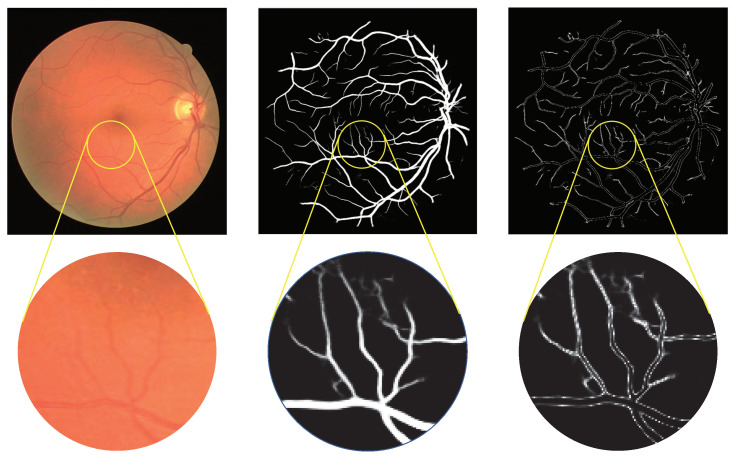
Schematic diagram of the effect of MFI module on vascular feature enhancement. The left subfigure shows the original image, and the middle portrays the output of the MVE module. The right subfigure displays the enhanced blood vessels after applying the MFI module, which enhances the details and edges of the vascular features.

After processing by this activation function, the pixels of the capillary vessel pixels and vascular boundaries, with a probability close to 0.5 in the feature maps [5], are improved to $1-e^{-0.5}$, and the pixels of the background and thick vessel declined to 0. Figure 5 shows the results produced by the activation function. As illustrated in the enlargement window, the pixels representing the capillaries and the edges of the thick vessel are maintained. Through utilizing the enhanced micro-vessel feature map to improve A/V classification, the impact caused by the imperfect vessel segmentation is alleviated.

### 3.4. Loss Function

MSC-Net is constrained by a loss function combined with vessel segmentation ($L_{v}$) and A/V classification ($L_{a}$). Based on the binary cross-entropy and the dice loss [27], $L_{v}$ is quantified as follows:

(5) $$L_{v}=L_{v}^{bce}+0.1L_{v}^{dice}$$

where $L_{v}^{bce}$ refers to the binary cross-entropy loss, $L_{v}^{dice}$ represents the dice loss for vessel segmentation, they are defined as:

(6) $$L_{v}^{bce}=\sum_{i}-\left[y_{i}log\left(p_{i}\right)+\left(1-y_{i}\right)log\left(1-p_{i}\right)\right]$$

(7) $$L_{v}^{dice}=1-\frac{2\sum y_{i}p_{i}+\epsilon }{\sum y_{i}+\sum p_{i}+\epsilon }$$

where $y_{i}$ refers to the label of the pixel, $p_{i}$ refers to prediction, and ϵ represents the smoothing factor setting as $10^{-6}$. Since the A/V classification is a multi-class classification task, the $L_{a}$ is quantified by a widely used cross-entropy function, as shown in (8).

(8) $$L_{a}=\sum_{i}\sum_{c=1}^{N}-y_{ic}log\left(p_{ic}\right).$$

where *N* is the number of categories, *c* refers to the index of a certain category, $y_{ic}$ represents whether the *i*th pixel belongs to the *c*th category, $p_{ic}$ refers to the prediction produced by the network.

The total loss is defined as

(9) $$Loss=L_{v}+L_{a}$$

## 4. Experimental Result

### 4.1. Datasets

**Figure 6:**
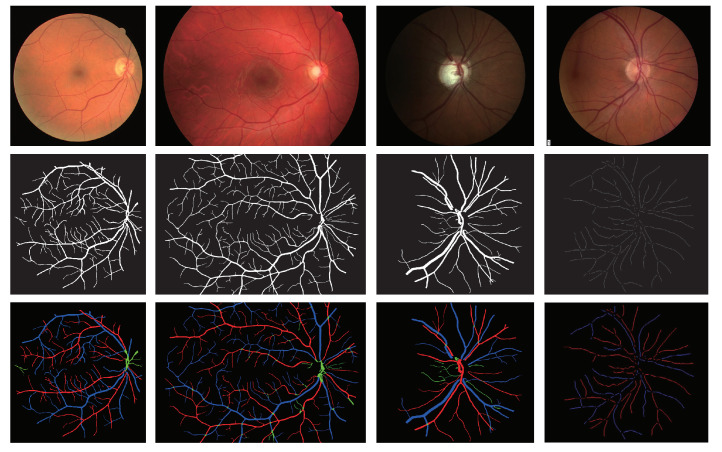
Sample images from DRIVE, HRF, LES, and INSPIRE datasets. The first column shows the original image, the vessel ground truth, and the A/V label of the DIRVE dataset sample from top to bottom. The second, third, and fourth columns show the samples from HRF, LES, and INSPIRE datasets.

To validate the performance of the methods, we employ four public datasets for experiments. Figure 6 shows some samples from the datasets.

#### 4.1.1. DRIVE

DRIVE (Digital Retinal Images for Vessel Extraction) dataset [28] is a classic retinal vessel segmentation dataset consisting of 40 images with a resolution of 584 × 565. In the original dataset, each image obtains a binary standard vessel segmentation label, where the vessel pixels are marked in white color. Based on [29], the vessel pixels are divided into three groups, including those referring to arteries, veins, and uncertain regions, labeled with red, blue, and green.

#### 4.1.2. HRF

HRF (High-Resolution Fundus) dataset is a high-resolution dataset containing 45 images with a huge resolution of 3504 × 2336 [30]. All the images belong to three categories, including images from healthy patients, glaucomatous patients, and diabetic patients. And each image owns a binary standard vessel segmentation label and an artery/vein classification label. This study selects one-third of each category as training data, while the remaining is used for testing and validation [5]. In other words, there are 15 images for training and 30 images for test and validation. Before training, all the images are resized to 1536 × 1024 pixels.

#### 4.1.3. LES

LES dataset includes 22 optic disc-centered images with a 30° field of view (FOV) and a resolution of 1620 × 1444 pixels [31]. Each image has an independent A/V classification result and blood vessel segmentation result. According to [5], select 11 pictures as training data, and use the rest for testing and validation. To better train and validate the model, all the images are resized to 1024 × 1024 pixels.

#### 4.1.4. INSPIRE

INSPIRE (the Iowa Normative Set for Processing Images of the Retina) dataset contains 40 optic disc-centered images, which with FOV of 30° and resolution of 2392 × 2048 pixels [32]. No pixel-wise vessel segmentation ground truth is available for the INSPIRE-AVR dataset and only the A/V classification of vessel centerline is provided. So, it is used to explore the generalization of MSC-Net. Before testing, all the images are resized to 1024 × 1024 pixels.

### 4.2. Preprocessing and Data Augmentation

**Figure 7:**
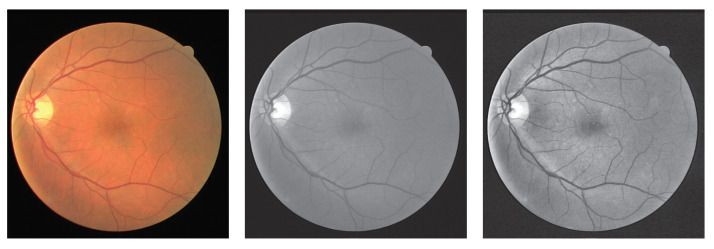
Results of preprocessing on the DRIVE dataset. The first column is the original image, the second column represents the green channel, and the last column shows the image processed by the CLAHE.

Before the training process, some augmentation methods are utilized to improve the amount of the training data and enhance the expression to alleviate the overfitting of the network. As a binary segmentation task, vessel segmentation needs more structural information about the vessel. Therefore, we utilize the single channel, the green channel of the original RGB image, to obtain the detailed vessel structure. Second, Contrast Limited Adaptive Histogram Equalization (CLAHE) [33] is employed to deal with the green channel to reduce the interference of color on blood vessel segmentation. The outputs of these operations are shown in Figure 7. For A/V classification, we use the original image to participate in the operation.

Then, the 256 × 256 pixels patches are randomly cropped from the training images. Random rotation and Gaussian noise addition are applied to these patches. For the test, each picture is cropped into several patches in order, and the vessel and A/V prediction is the connection of all the patches results related to one image.

We implemented the proposed model using Python, leveraging the PyTorch deep learning framework, and trained and tested it on an NVIDIA TITAN Xp graphics card with 12 GB of RAM, optimizing the model parameters with the Adam optimizer using 100 iterations and a learning rate of 0.001.

### 4.3. Evaluation Metrics

The metrics accuracy (Acc), sensitivity (Sen), specificity (Spe), and F1-score (F1) are used to evaluate the performance of the network. For A/V classification, these metrics are turned to $Acc_{a}$, $Sen_{a}$, $Spe_{a}$, and $F1_{a}$, while when they measure the performance of vessel segmentation, they are marked with $Acc_{v}$, $Sen_{v}$, $Spe_{v}$, and $F1_{v}$. All the metrics are calculated as follows:

(10) $$Spe=\frac{TN}{TN+FP}$$

(11) $$Sen=\frac{TP}{TP+FN}$$

(12) $$Acc=\frac{TP+TN}{TP+FP+TN+FN}$$

(13) $$F1=\frac{2\times TP}{2\times TP+FP+FN}$$

The parameters’ true positives (TP) and true negatives (TN) measure the quantity of correctly classified positives and negatives, respectively, while false positives (FP) and false negatives (FN) relate to the number of false classifications.

For A/V classification, the positives describe the artery pixels, whereas the negatives describe the vein pixels. Sensitivity ($Sen_{a}$) measures the algorithm’s ability to detect arteries, while specificity ($Spe_{a}$) assesses the method’s ability to detect veins. Accuracy ($Acc_{a}$) represents the algorithm’s capacity to differentiate between arteries and veins, while the F1 score ($F1_{a}$) represents the overall accuracy of the algorithm.

For vessel segmentation, the positives refer to vessel pixels, and the negatives refer to background pixels. Sensitivity ($Sen_{v}$) only relates to whether the algorithm correctly classifies vessel pixels, whereas specificity ($Spe_{v}$) only relates to background pixels. Accuracy ($Acc_{v}$) and F1 score ($F1_{v}$) demonstrate the algorithm’s overall accuracy in separating vessel pixels from background pixels in different directions.

### 4.4. Ablation Studies

We conduct ablation studies on the DRIVE dataset for A/V classification and vessel segmentation to validate the contribution of each module in MSC-Net. To demonstrate the contribution of modules to the classification and segmentation tasks more effectively, we choose the double U-Net as the baseline network, which obeys the same strategy as MSC-Net. In this network, the first U-Net extracts the vessel features, and the second U-Net produces the A/V result. In the following experiments, we regard the U-like network with the first downsample depth of 64 as the original U-Net.

Table 1 illustrates a detailed summary of the performance of various module combinations on the DRIVE dataset. The performance of networks is greatly improved with the utilization of the MVE, MAE, and MFI modules. Compared to single-task networks, which are summarized in the first two rows in Table 1, multi-task networks exhibit higher performance evaluation metrics, with the baseline network even surpassing the single-task networks. Due to the replacement of the original U-Net with the MVE module, the baseline network achieves an improvement of 0.0126, 0.0024, 0.0007, and 0.0036 on $Sen_{v}$, $Spe_{v}$, $Acc_{v}$, and $F1_{v}$, revealing that the MVE module could extract more critical information about vessel structures than the original U-Net, which is beneficial for the A/V classification. Moreover, with the alternation of the original U-Net in the baseline network with the MAE module, the performance of the baseline network is improved by $Sen_{a}$ of 0.0155, $Spe_{a}$ of 0.0045, $Acc_{a}$ of 0.011, and $F1_{a}$ of 0.0111, demonstrating that the novel MAE module could significantly improve the network’s performance compared to the original U-Net by capturing long-range dependencies between pixels. Additionally, the baseline network achieves improved performance by combining the MFI module, which effectively fuses different feature representations to enhance network performance. After adding this module, all the metrics of the baseline network have improved, especially the $Sen_{a}$ increased by 0.0178, $Acc_{a}$ by 0.0112, and $Sen_{v}$ by 0.0278. It is noteworthy that the MSC-Net combining three modules achieves the best results with $Sen_{a}$ of 0.9494, $Spe_{a}$ of 0.9440, $Acc_{a}$ of 0.9469, and $F1_{a}$ of 0.9398, which denote the superior performance of the MSC-Net on A/V classification task. The highest metrics with $Sen_{v}$ of 0.8527, $Spe_{v}$ of 0.9785, $Acc_{v}$ of 0.9685, and $F1_{v}$ of 0.8107 demonstrate that the combination of the proposed three modules could also obtain the high-quality vessel segmentation results.

**Figure 8:**
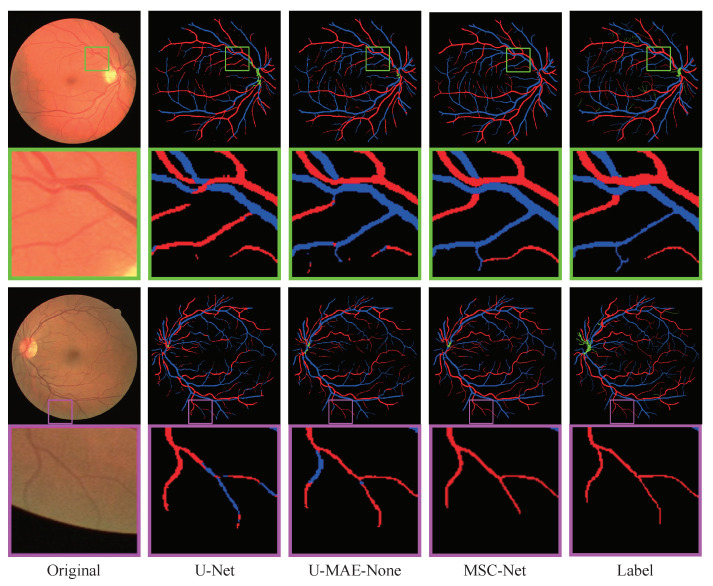
The processing results of ablation studies.

Furthermore, in Figure 8, we compared the detailed visualization of the A/V classification results of the baseline, the alternation network that replaced the U-Net in the baseline network by the MAE module, and MSC-Net, in which some specific regions are highlighted with different colors. From the figure, we can see that, after the alternation of the original U-Net with the MAE module, the performance of the baseline network extracting tiny vessels is improved. Moreover, with the addition of the MVE and MFI modules, the enhanced vascular maps will preserve the microscopic structures in the original image, which is beneficial for the A/V classification. As shown in the green box of Figure 8, facing the vessel cross areas, the baseline network misclassifies the intersection category, while the alternation network and MSC-Net perform well. Moreover, in the purple box, the baseline and alternation network both confuse the belonging of the ends of the vessel. On the other hand, the MSC-Net, which combined the MVE, MAE, and MFI modules, could give a high-quality and correct classification result.

### 4.5. Comparison with Existing Methods

The comparison results on vessel segmentation and A/V classification are shown in Table 2 and Table 3. The methods employed for comparison experiments are classified into the methods based on hybrid structures [12,34,35] and the convolution methods combined with some graph methods [11,36,37,38]. The comparison results of A/V classification between the proposed method and other state-of-the-art methods on the DRIVE and HRF datasets are summarized in Table 3. For the DRIVE dataset, our MSC-Net achieves the optimal $Sen_{a}$ of 0.9494, $Spe_{a}$ of 0.9441, $Acc_{a}$ of 0.9469, and $F1_{a}$ of 0.9398. The results are, respectively, 0.0194, 0.0111, 0.0128, and 0.0208 higher than that of the existing best results [12,34,35,36]. Moreover, in addition to the commonly used DRIVE dataset, we evaluate the proposed method on the HRF and LES datasets, which have a higher resolution and more complicated fundus conditions. As listed in Table 3, our method demonstrates better extraction and reorganization capabilities with the best $Sen_{a}$ of 0.9720, $Spe_{a}$ of 0.9758, and $Acc_{a}$ of 0.9735 on HRF dataset, and the best $Sen_{a}$ of 0.9030, $Spe_{a}$ of 0.9155, $Acc_{a}$ of 0.9072, and $F1_{a}$ of 0.8947 on LES dataset.

Furthermore, we also compare the vessel segmentation performance of MSC-Net against other state-of-the-art methods. In Table 2, we summarize the comparison results of different methods on DRIVE, HRF, and LES datasets. From the table, it can be confirmed that the proposed method has achieved the best performance on all of the DRIVE, HRF, and LES datasets. MSC-Net achieves the highest $Sen_{v}$ of 0.8527, $Acc_{v}$ of 0.9684, $F1_{v}$ of 0.8107, and the comparable $Spe_{v}$ of 0.9785 on the DRIVE dataset, reflecting its superior feature extraction capability in identifying tiny structures such as micro-vessel pixels. For the HRF dataset, the best $Sen_{v}$ of 0.8578 and $Acc_{v}$ of 0.9704 showcases the effectiveness of the proposed method in precisely segmenting the vessels, even in images with high-resolution conditions and complex vessel tree structures. On the LES dataset, the highest $Sen_{v}$ of 0.8824, $Spe_{v}$ of 0.9885, $Acc_{v}$ of 0.9825, and $F1_{v}$ of 0.8518 demonstrate the superior performance of the proposed method on the more complicated fundus conditions.

**Figure 9:**
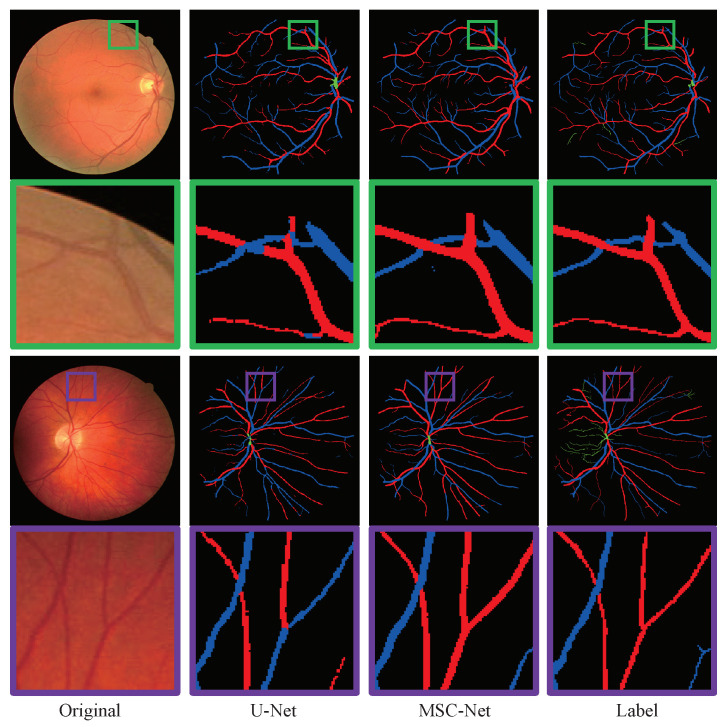
The A/V classification results on images from the DRIVE dataset.

**Figure 10:**
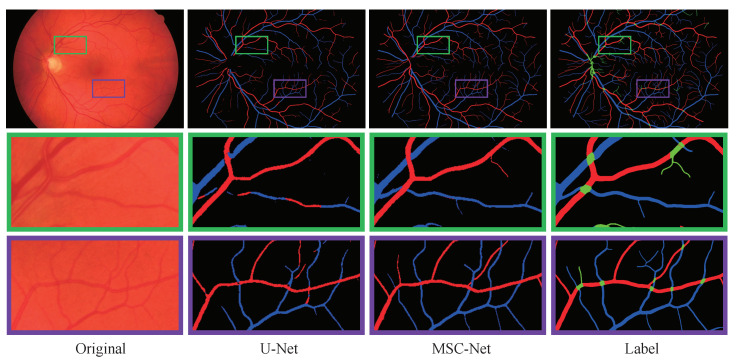
The A/V classification results on images from the HRF dataset.

**Figure 11:**
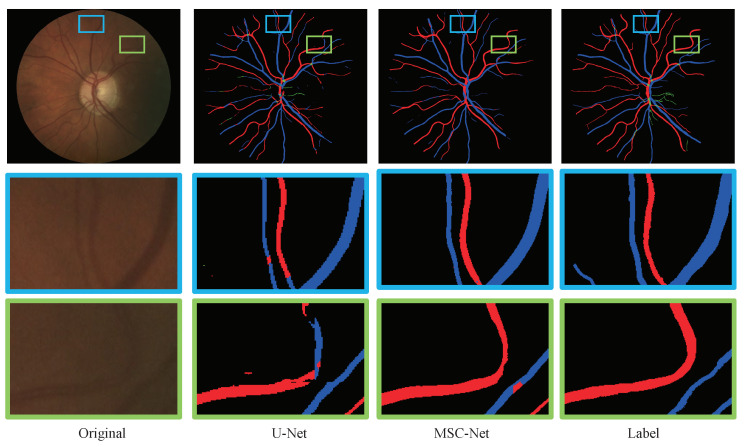
The A/V classification results on images from the LES dataset.

Additionally, some sample images are selected from the DRIVE, HRF, and LES datasets. Their A/V classification predictions, generated by the original U-Net or the proposed method, are presented in Figure 9, Figure 10 and Figure 11. In Figure 9, it is evident that MSC-Net is more robust than U-Net in identifying vessel crossing areas and the bifurcation of blood vessels. Specifically, MSC-Net could correctly identify the vessel crossing areas in the green box, which presents a challenge for U-Net. Similarly, MSC-Net classifies the primary and secondary vessels into appropriate categories in the purple box, whereas U-Net encounters difficulties in accurately classifying them. Furthermore, as shown in Figure 10 and Figure 11, the MSC-Net obtains higher quality results for high-resolution fundus images than the U-Net. Based on the visualization results, the proposed MSC-Net could extract tiny vessels from the original image and efficiently classify the arteries and veins, demonstrating its superior capability in detail extraction.

To enhance the robustness of the proposed model, fusing datasets with different scales can enrich the quantity and distribution of training data, which can be validated on a new dataset with multiscale. This approach can effectively address the issue of limited data availability and distribution, providing a broader range of representative samples for model training. As shown in Table 4, the first three rows are only trained on the DRIVE, HRF, or LES datasets and tested on the INSPIRE dataset. Moreover, the last row is trained on the combination of the above three datasets. From the table, the best results are obtained in the last row, showing that fusing datasets with different scales can enhance the robustness of the model.

## 5. Conclusions

In this paper, we proposed a novel deep learning network, named the Multi-task Segmentation and Classification Network. The network deploys a novel strategy, which utilizes the results of vessel segmentation produced by the Multi-scale Vessel Extraction module to enhance the A/V classification. The proposed Multi-scale Vessel Extraction module employs a series of tiny-kernel convolution blocks adequately to reduce the influence of background pixels and obtain multi-scale vessel features. The enhanced image contains a lot of structural information belonging to the original image, which is beneficial to the A/V classification. The newly designed MAE module utilizes the hybrid structure of adjusted transformer-like and convolution blocks to learn and classify the different pixels. The MFI module could fuse the outputs of the former two modules to obtain the high-quality A/V classification results and vessel segmentation results, simultaneously.

Due to the concatenation of the original image and the output of the Multi-scale Vessel Extraction module, the Multi-structure A/V Extraction module obtains more information about the tiny structures from the input data, which could help the network better accomplish the task of A/V classification. At the same time, with the addition of the Multi-source Feature Integration module, the insurmountable limitation of the two-stage method is alleviated. Experimental results show that our method is superior to the existing state-of-the-art methods on all of the tested public datasets.

## Figures and Tables

**Table 1:** Results of the ablation studies for A/V classification on DRIVE dataset (Key: **Best**).

Methods				A/V Classification					Vessel Segmentation			
MVE/U	MAE/U	MFI		${\mathrm{Sen}}_{a}$	${\mathrm{Spe}}_{a}$	${\mathrm{Acc}}_{a}$	$F1_{a}$		${\mathrm{Sen}}_{v}$	${\mathrm{Spe}}_{v}$	${\mathrm{Acc}}_{v}$	$F1_{v}$
-/✓	-/-	-		-	-	-	-		0.8019	0.9713	0.9541	0.8002
-/-	-/✓	-		0.9242	0.9160	0.9200	0.9098		-	-	-	-
-/✓	-/✓	-		0.9229	0.9304	0.9273	0.9202		0.8213	0.9757	0.9655	0.8053
✓/-	-/✓	-		0.9231	0.9383	0.9309	0.9332		0.8339	0.9781	0.9662	0.8089
-/✓	✓/-	-		0.9384	0.9349	0.9383	0.9313		0.8309	0.9774	0.9662	0.8074
✓/-	✓/-	-		0.9460	0.9389	0.9438	0.9363		0.8418	0.9775	0.9673	0.8100
-/✓	-/✓	✓		0.9407	0.9352	0.9385	0.9278		0.8491	0.9779	0.9673	0.8052
✓/-	-/✓	✓		0.9466	0.9346	0.9433	0.9267		0.8435	0.9784	0.9684	0.8098
-/✓	✓/-	✓		0.9471	0.9398	0.9436	0.9360		0.8519	0.9782	0.9682	0.8090
✓/-	✓/-	✓		**0.9494**	**0.9440**	**0.9469**	**0.9398**		**0.8527**	**0.9785**	**0.9685**	**0.8107**

U, Original U-Net.

**Table 2:** Results of the comparison experiments for vessel segmentation (Key: **Best**).

Dataset	Methods	${\mathrm{Sen}}_{v}$	${\mathrm{Spe}}_{v}$	${\mathrm{Acc}}_{v}$	$F1_{v}$
DRIVE	U-Net [6]	0.8019	0.9713	0.9541	0.8002
	AC-Net [35]	0.7916	**0.9811**	0.9570	N/A
	CE-Net [39]	0.7903	0.9769	0.9550	N/A
	SA-Unet [40]	0.8112	0.9767	0.9641	0.8027
	**MSC-Net**	**0.8527**	0.9785	**0.9684**	**0.8107**
HRF	U-Net [6]	0.8319	0.9790	0.9610	0.7987
	UA-Net [12]	0.8500	0.9100	0.9100	0.6200
	VC-Net [5]	0.7903	**0.9843**	0.9663	**0.8101**
	**MSC-Net**	**0.8578**	0.9785	**0.9704**	0.8001
LES	U-Net [6]	0.8595	0.9808	0.9748	0.7755
	UA-Net [12]	0.8504	0.9840	0.9722	0.8417
	**MSC-Net**	**0.8824**	**0.9885**	**0.9825**	**0.8518**

N/A, Not Available.

**Table 3:** Results of the comparison experiments for A/V classification (Key: **Best**).

Dataset	Methods	${\mathrm{Sen}}_{a}$	${\mathrm{Spe}}_{a}$	${\mathrm{Acc}}_{a}$	$F1_{a}$
DRIVE	U-Net [6]	0.9242	0.9160	0.9200	0.9098
	Li et al. [36]	0.9000	0.8400	0.9190	0.9190
	Girard et al. [38]	0.8630	0.8680	0.8650	N/A
	Noh et al. [34]	0.9300	0.9220	0.9260	N/A
	AC-Net [35]	0.9220	0.9330	0.9260	N/A
	AV-Net [11]	0.8863	0.9272	0.9081	N/A
	UA-Net [12]	0.8900	0.9250	0.9341	0.8800
	**MSC-Net**	**0.9494**	**0.9441**	**0.9469**	**0.9398**
HRF	U-Net [6]	0.9274	0.9178	0.9570	0.9714
	VC-Net [5]	N/A	0.9588	0.9704	N/A
	UA-Net [12]	0.9120	0.9013	0.9613	**0.9769**
	**MSC-Net**	**0.9720**	**0.9758**	**0.9735**	0.9717
LES	U-Net [6]	0.8640	0.9044	0.8771	0.8885
	UA-Net [12]	0.8800	0.8588	0.8604	N/A
	**MSC-Net**	**0.9030**	**0.9155**	**0.9072**	**0.8947**

**Table 4:** The model is trained under the selected training dataset and tested under the INSPIRE dataset (Key: **Best**).

Training Datasets				A/V Classification			
DRIVE	HRF	LES		${\mathrm{Sen}}_{a}$	${\mathrm{Spe}}_{a}$	${\mathrm{Acc}}_{a}$	$F1_{a}$
✓	-	-		0.6873	0.6470	0.6686	0.7151
-	✓	-		0.8475	0.7511	0.8045	0.7714
-	-	✓		0.8640	0.6461	0.7048	0.6385
✓	-	-		**0.8742**	**0.8632**	**0.8656**	**0.8565**

## Data Availability

Not applicable.
